# Visceral symptoms in patients with anterior cutaneous nerve entrapment syndrome (ACNES): expression of viscerosomatic reflexes?

**DOI:** 10.1007/s10029-023-02827-7

**Published:** 2023-07-01

**Authors:** Monica L. Y. E. Jacobs, Loes Janssen, Laurents P. S. Stassen, Marc R. M. Scheltinga, Rudi M. H. Roumen

**Affiliations:** 1 Department of Surgery, Máxima Medical Centre, Veldhoven, The Netherlands; 2 Centre of Expertise for ACNES, Centre of Excellence for Abdominal Wall and Groin Pain, Máxima Medical Centre, SolviMáx, Eindhoven, The Netherlands; 3 Department of Surgery, Maastricht University Medical Centre (MUMC), Maastricht, The Netherlands; 4 Department of Surgery and NUTRIM, Maastricht University, Maastricht, The Netherlands

**Keywords:** Abdominal wall, Chronic abdominal pain, Visceral symptoms, Viscera, Viscerosomatic reflexes

## Abstract

**Purpose:**

Anterior cutaneous nerve entrapment (ACNES) is characterized by neuropathic pain in a predictable, circumscript abdominal area. The diagnostic delay is long, with half of ACNES-affected individuals reporting nausea, bloating, or loss of appetite mimicking visceral disease. The aim of this study was to describe these phenomena and to determine whether treatment could successfully reverse the visceral symptoms.

**Methods:**

This prospective observational study was conducted between July 2017 and December 2020 at SolviMáx, Center of Excellence for Chronic Abdominal Wall and Groin Pain, Máxima Medical Center, Eindhoven. Adult patients who fulfilled published criteria for ACNES and reported at least one visceral symptom at intake were eligible for the study. A self-developed Visceral Complaints ACNES Score (VICAS) questionnaire that scores several visceral symptoms (minimum 1 point, maximum 9 points) was completed before and after therapy. The success of treatment was defined as at least 50% reduction in pain.

**Results:**

Data from 100 selected patients (86 females) aged 39 ± 5 years were available for analysis. Frequently reported symptoms were abdominal bloating (78%), nausea (66%) and altered defecation (50%). Successful treatment significantly reduced the number of visceral symptoms, with a VICAS before of 3 (range 1–8) and after of 1 (range 0–6) (*p* < 0.001). A low baseline VICAS was associated with successful treatment outcome (OR 0.738, 95% CI 0.546–0.999).

**Conclusion:**

Patients with ACNES may report a variety of visceral symptoms. Successful treatment substantially reduces these visceral symptoms in selected patients.

## Introduction

A potential source of chronic abdominal pain that is often neglected is the abdominal wall itself [1]. A typical example of a chronic abdominal wall pain condition is anterior cutaneous nerve entrapment syndrome (ACNES) [2, 3]. ACNES is thought to be due to compromised intercostal nerve endings that originate from the 7th to 12th thoracic vertebrae [4, 5]. These nerves penetrate the rectus abdominis muscle and innervate the overlying skin. ACNES often presents as severe unilateral pain in a predictable area of the anterior abdomen that harbors altered skin sensations, such as hypoesthesia, hyperesthesia, and changed cool perception [3]. Squeezing the skin and subcutaneous tissue between the thumb and index finger in this area (pinch test) is disproportionally painful. Located centrally in this area is a spot of maximal tenderness (tender point, TP) that is Carnett positive, i.e., increased pain when simultaneously tensing the rectus abdominis muscle and palpating the painful area [4]. Pain reduction after local injection of an anesthetic agent at the TP supports a diagnosis of ACNES.

A large population study (*n* = 1116 patients) found that the median diagnostic delay for ACNES was 18 months [6]. The long delay is likely because approximately half of painful ACNES patients also report a range of visceral symptoms such as nausea, bloating, or loss of appetite mimicking visceral entities [6]. This high proportion may contribute to many ACNES patients being wrongfully diagnosed with having a functional abdominal pain syndrome [7]. Such patients are evaluated not only by general practitioners, but also by internal medicine physicians and gastro-enterologists.

Visceral symptoms in ACNES may be an expression of so-called *segmental* phenomena. For instance, bloating, or the feeling of abdominal distension, may be caused by abnormal viscerosomatic reflexes and may contribute to actual girth distension [8]. ACNES may, therefore, reflect a compromised segmental relation between a viscus and the corresponding somatic nerve, resulting in a painful head zone. This zone is a somatic nerve dermatome with altered skin sensation and painful spots associated with a viscus at an advanced stage of disease [9]. For example, hyperesthesia of the T10–11 dermatome in the right lower abdominal area may reflect acute appendicitis [10].

The aim of the present study is to gain more insight into the visceral complaints among ACNES patients and the effect of treatment on these complaints.

## Methods

### Setting and design

This prospective observational study was conducted between July 2017 and December 2020 at the Center of Excellence for Chronic Abdominal Wall and Groin Pain. Clinicians at this institute have gained vast experience over the past 15 years in the treatment of chronic abdominal wall pain syndromes, including ACNES [11]. Patients referred for evaluation of chronic abdominal pain are first requested to complete a standard intake questionnaire that includes demographics, history and other specifics. Each patient suspected of having ACNES is then invited to our outpatient department for evaluation by a dedicated team of surgeons, physicians and physician assistants. For the purpose of this study, consecutive patients were recruited by one of the senior authors (RR or MS), but treated by the entire team of physicians. To avoid bias, the first author only performed study procedures after the patients had been treated. All patients had given informed consent prior to intake, and the study protocol was approved by the Medical Ethics Committee of our hospital (M17.115).

### Patient assessment

Study criteria were the presence of previously reported diagnostic criteria for ACNES [12, 13]:

- predictable site of abdominal tenderness with a small (< 2 cm diameter) area of maximal intensity (tender point, TP) located within the lateral boundaries of the rectus abdominis muscle
- unilateral or bilateral TP
- presence of somatosensory skin disturbances covering the TP, such as altered cool sensation, hypoesthesia, or hyperesthesia
- intense pain while squeezing the abdominal wall skin covering the TP (pinch test)
- increased tenderness after abdominal muscle tensing with (Carnett’s test)

Only ACNES patients who reported at least one visceral symptom related to the perceived abdominal pain during the intake consultation were eligible for entry to the study. Exclusion criteria were recently treated elsewhere for ACNES, recent intra-abdominal pathology, lidocaine allergy, pregnancy, and language barrier.

### Management of ACNES

If the patient was diagnosed with ACNES, a freehand subfascial injection of 1% lidocaine (5–10 mL) was administered just beneath the anterior rectus sheath at the TP, as reported previously [14]. Three consecutive injection sessions were scheduled for the following 6 weeks. If the residual level of pain was acceptable, subsequent injections were canceled [15]. If pain reduction was insufficient after this regimen, pulsed radio frequency (PRF) or surgical removal of the nerve (anterior neurectomy) was proposed [16]. The various steps in this treatment strategy were determined in a shared decision environment with the patient.

### Visceral Complaints ACNES Score

A Visceral Complaints ACNES Score (VICAS) questionnaire in Dutch language was developed by the senior authors based on their subjective, long-term experience with ACNES treatment. A total of 9 visceral symptoms related to the pain were scored. Each patient was asked whether a visceral symptom was present (yes, 1 point) or absent (no, 0 point), thus giving a minimum score of 1 (just one visceral symptom) and a maximum score of 9 points (all visceral symptoms present; Table 1). Belching was defined as the process of releasing accumulated air in the stomach by the mouth, thereby relieving distension or discomfort. Retching was the sound and/or movement of vomiting without actually vomiting. Abdominal bloating was the subjective feeling of an increased size of the abdomen. Urinary tract complaints could be due to a variety of symptoms and were not well predefined. The questionnaire was completed by participants before treatment, and again at least three months after the final treatment step.

**Table 1:** Questionnaire scoring visceral symptoms in ACNES (minimum 1 point, maximum 9 points), English translation

9-item Visceral Complaints ACNES Score (VICAS)	
Symptom	Yes (1)/no (0)
Belching	
Retching	
Increased postprandial pain	
Loss of appetite	
Nausea	
Abdominal bloating	
Altered defecation	
Increased flatulency	
Urinary tract complaints	
Total score	/9

### Outcome measures

Baseline characteristics such as length, weight, numeric rating scale for pain (score of 0–10, where 0 = no pain and 10 = worst pain) were obtained from the completed questionnaires and electronic patient files. The main outcomes were the frequency and the number (VICAS) of various visceral symptoms prior to treatment in ACNES patients. Next, the relationship between the presence of visceral symptoms and the outcome from ACNES treatment was evaluated. Treatment outcome was based on pain reduction. A four-point patient satisfaction score for pain reduction was obtained from the options shown below. This was obtained by telephone by the first author after completion of the final treatment step, as reported previously [14]:

1. I am very satisfied (> 95% pain reduction)
2. I am satisfied, but I occasionally experience some pain (≥ 50% pain reduction)
3. I have improved, but experience some pain on a regular basis (< 50% pain reduction)
4. The treatment did not change my pain level, or made my pain worse

Treatment outcome was considered successful if the patient’s response was category 1 or 2, and unsuccessful if it was 3 or 4. If the patient did not respond after two telephone attempts, the questionnaire was sent by mail. If this questionnaire was not returned, the patient was considered as lost to follow-up.

### Statistical methods

An arbitrary sample size of 100 completed questionnaires was targeted a priori. Anticipating a 10% drop-out rate, 110 patients were needed. Continuous data were presented as the mean and standard deviation (SD) if parametric, and median and range if non-parametric. Categorical variables were presented as a count and percentage. Continuous variables were analyzed using the *t* test for parametric variables, and the Mann–Whitney *U* test (unpaired) or Wilcoxon signed-rank test (paired) for non-parametric variables. Categorical variables were analyzed using the McNemar test, Pearson Chi-square test, or Fisher’s exact test as appropriate. Univariate logistic regression analysis was used to determine whether baseline VICAS or patient characteristics were predictive of the overall treatment outcome. Patient characteristics associated with treatment outcome (i.e., *p* < 0.1) were entered into a multivariate analysis together with VICAS. This was used to study the effect of baseline VICAS on treatment outcome, while accounting for possible confounders. A *p* value ≤ 0.05 was considered to represent statistical significance. Data analysis was performed using SPSS version 23 for Windows.

## Results

**Fig. 1:**
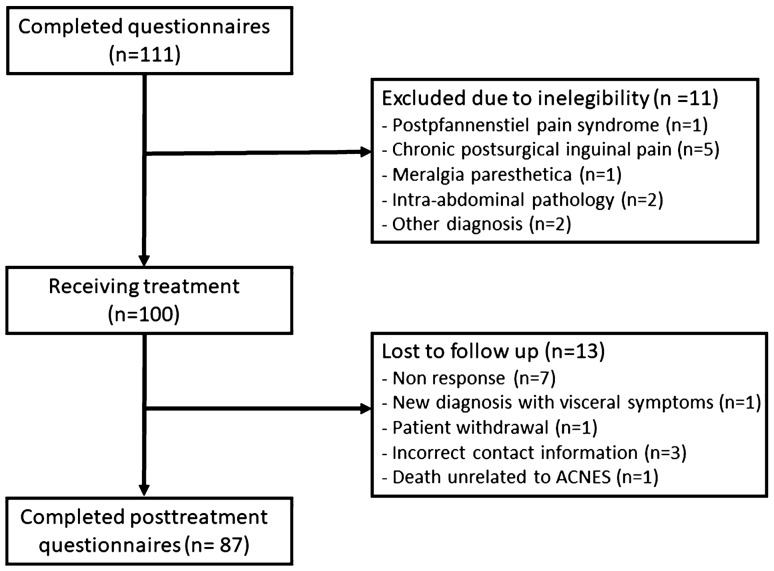
Flow chart of ACNES patients presenting with pain and visceral symptoms

A total of 111 patients from July 2017 to August 2018 fulfilled the study criteria. Eleven patients were excluded due to ineligibility, giving a final cohort of 100 patients for the analysis (Fig. 1). Of these, 13 patients were lost to follow-up, giving 87 patients in whom evolution of visceral symptoms could be determined. Baseline characteristics for the patients are presented in Table 2.

**Table 2:** Characteristics of selected ACNES patients presenting with one or more visceral symptoms (*n* = 100)

Age*	39 (18)
Female	86 (86%)
Height (cm)*	170 (10)
Weight (kg)*	74 (17)
BMI (kg/m^2^)*	25 (5)
NRS pain (0–10) †	7 (1–9)
Abdominal wall TP location	
Right upper quadrant (T7–9)	11 (11%)
Right lower quadrant (T10–12)	38 (38%)
Left upper quadrant (T7–9)	17 (17%)
Left lower quadrant (T10–12)	9 (9%)
Bilateral TP	19 (19%)
Multiple TPs	6 (6%)
Analgesic use	
Paracetamol	36 (36%)
NSAID	20 (20%)
Opioids	25 (25%)
Neuroleptics	5 (5%)
Previous abdominal history	
Irritable bowel syndrome	18 (18%)
Diverticulosis	4 (4%)
Bariatric surgery	3 (3%)
Endometriosis	3 (3%)
Constipation	3 (3%)
Inflammatory bowel disease	1 (1%)
Treatment regimen	
Injection regimen	99 (99%)
Pulsed Radiofrequency	44 (44%)
Anterior neurectomy	44 (44%)
Posterior (2nd) neurectomy	28 (28%)
Other	4 (4%)

*BMI* body mass index, *NRS* Numeric Rating Scale, *TP* tender point, *T7* thoracic 7 nerve, *NSAID* Non Steroid Anti Inflammatory Drug

Data are presented as *means with standard deviations, †median and range, or number with percentages

### Visceral symptoms

The frequencies of visceral symptoms in ACNES patients are shown in Table 3. A median of three visceral symptoms (range 1–8) was reported. Apart from the obligatory abdominal pain, the other most frequent visceral complaints were bloating, nausea and altered defecation.

**Table 3:** Visceral symptom frequency among 100 ACNES patients

Abdominal bloating	78%
Nausea	66%
Altered defecation	50%
Increased postprandial pain	40%
Increased flatulency	38%
Loss of appetite	36%
Belching	25%
Urinary tract complaints	19%
Retching	17%

### Visceral symptoms in relation to tender point location

**Fig. 2:**
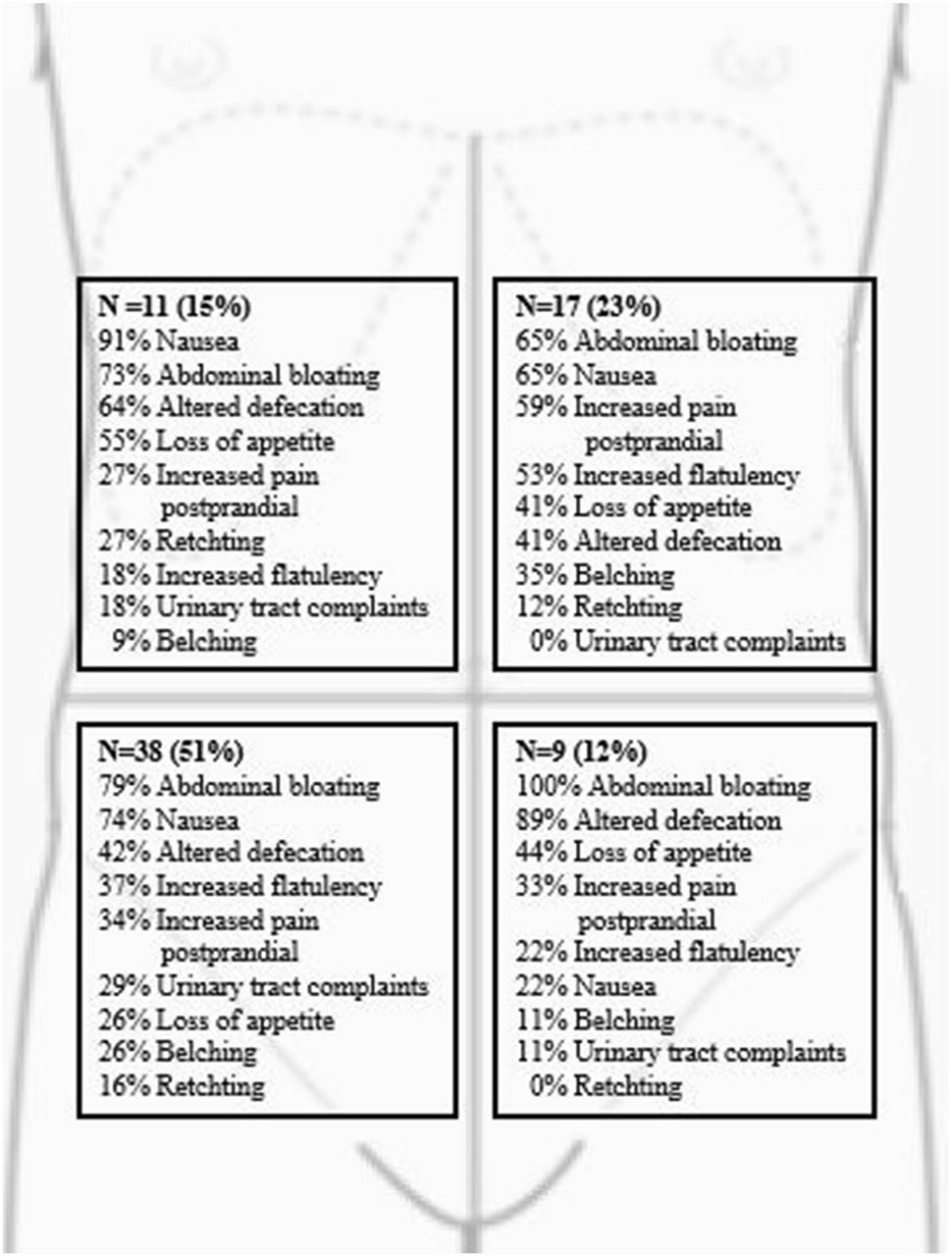
Frequency of visceral symptoms in relation to location in 75 unilateral ACNES patients

The relation between TP location and type of visceral symptom was studied in patients with a unilateral ACNES (*n* = 75; Fig. 2). Figure 2 shows the distribution of the various symptoms. Half the patients (51%) had a TP in the right lower abdominal quadrant. No pattern of preferred symptoms was seen in any of the abdominal quadrants.

### Relation of visceral symptoms and ACNES treatment outcome

The changes in visceral symptoms following treatment are shown in Table 4. Successful treatment significantly reduced 7 of 9 visceral symptoms, as reflected also by the fall in median VICAS from 3 before treatment to 1 afterwards (*p* < 0.001). Visceral symptoms did not change in patients who reported an unsuccessful outcome (*p* = 0.777). Before treatment, the median VICAS was 3 in the successfully treated group and 5 in the unsuccessful group (*p* = 0.005).

**Table 4:** Visceral symptoms before and after successful or failed treatment for ACNES

	Successful (*n* = 68)			Failed (*n* = 19)		
	Before	After	*p* value	Before	After	*p* value
Abdominal bloating	49 (72%)	31 (46%)	**0.001**	18 (95%)	17 (90%)	1.000
Nausea	46 (68%)	10 (15%)	** < 0.001**	13 (68%)	14 (74%)	1.000
Altered defecation	35 (52%)	14 (21%)	** < 0.001**	12 (63%)	12 (63%)	1.000
Increased pain postprandial	22 (32%)	9 (13%)	**0.007**	12 (63%)	12 (63%)	1.000
Increased flatulency	26 (38%)	15 (22%)	**0.052**	8 (42%)	10 (53%)	0.625
Loss of appetite	25 (37%)	5 (7%)	** < 0.001**	8 (42%)	8 (42%)	1.000
Belching	17 (25%)	7 (10%)	**0.006**	6 (32%)	3 (16%)	0.375
Urinary tract complaints	10 (15%)	7 (10%)	0.508	5 (26%)	6 (32%)	1.000
Retching	8 (12%)	0 (0%)	0.227	5 (26%)	1 (5%)	0.687
VICAS, median (range)	3 (1–8)	1 (0–6)	** < 0.001**	5 (2–7)	4 (1–8)	0.777

Bold values indicate statistical significance (*p* value ≤ 0.05)

**Fig. 3:**
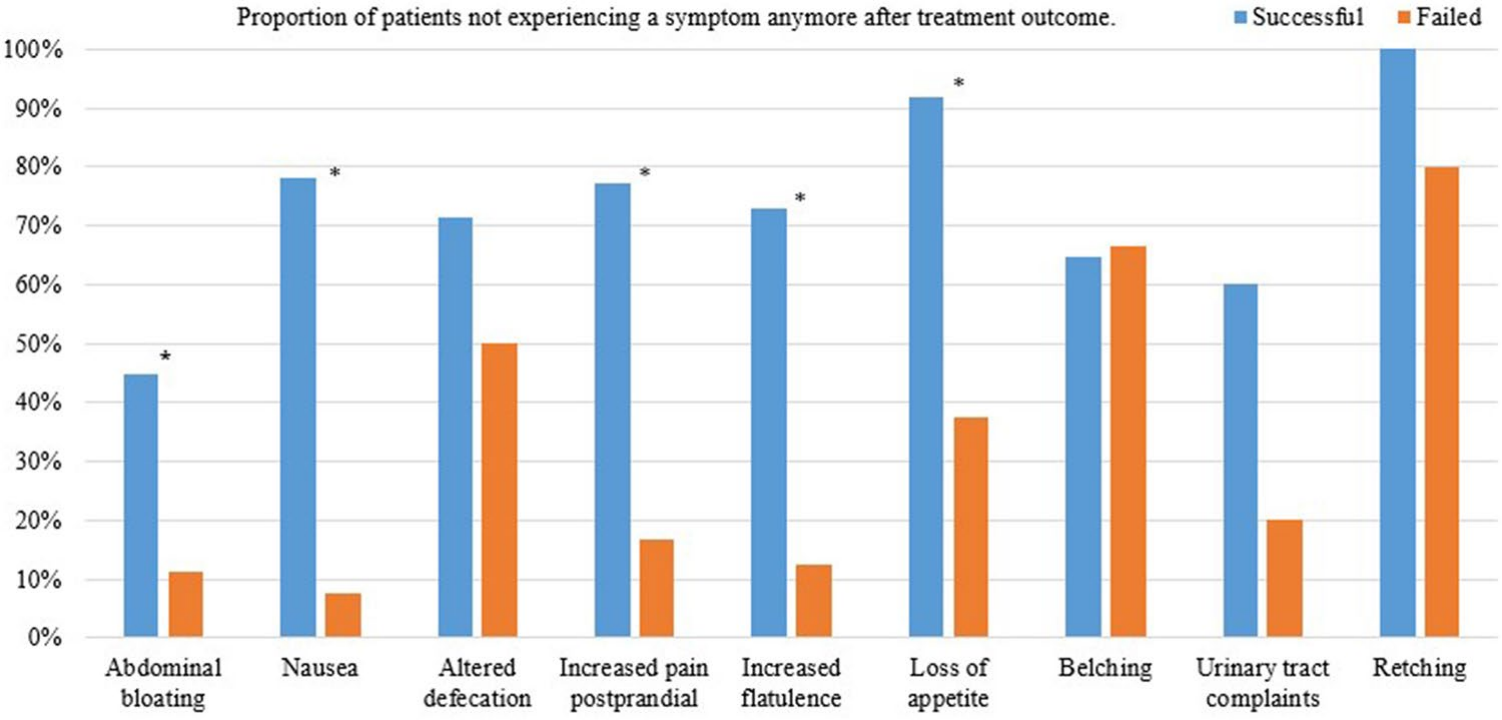
Proportion of patients no longer experiencing a visceral symptom in relation to the treatment outcome. *Significant difference, *p* value ≤ 0.05

Figure 3 shows the proportion of patients who reported the disappearance of an earlier visceral symptom after successful or failed treatment. For example, of the patients who experienced nausea before treatment, this condition disappeared in 78% of successfully treated patients but in just 8% of those who were unsuccessfully treated.

### Predictive factors for ACNES treatment outcome

A low baseline VICAS was significantly associated with the successful treatment of ACNES (OR 0.738, 95% CI 0.546–0.999). However, patient gender, age, baseline NRS and bilateral TP location were not predictive of treatment outcome and, therefore, multivariate analysis was not indicated.

## Discussion

The aim of this study was to describe the spectrum of visceral symptoms experienced by patients diagnosed with ACNES. Although pain is a hallmark of ACNES, approximately 50% of patients also experience a range of visceral complaints such as bloating or nausea [6]. This correlation has not been described in detail in the previous literature on ACNES. The present results show that selected ACNES patients report a median of three visceral symptoms. Moreover, successful ACNES treatment reversed most of these symptoms. Our results indicate that treatment failure for ACNES is related to the number of visceral symptoms. To the best of our knowledge, this is the first study describing and evaluating the specific relation between abdominal wall pain and visceral symptoms.

The presence of visceral symptoms in patients suffering from ACNES is potentially misleading, since this suggests a visceral origin. This presentation often leads to delayed or incorrect diagnosis, futile investigations, and excessive medical costs. Abdominal bloating, nausea and altered defecation are the most frequently reported visceral symptoms in ACNES. However, these are often also experienced by patients with irritable bowel syndrome (IBS). For example, 3.6% of patients diagnosed with IBS were instead found to have ACNES [7]. ACNES should, therefore, be considered in the differential diagnosis of abdominal pain in patients with various visceral symptoms. A previously published, 18-item chronic abdominal wall pain scoring list clearly distinguished an abdominal wall-related pain entity from a more functional pain syndrome, including IBS [17]. In our experience, ACNES patients with such gastro-intestinal complaints are often evaluated initially by gastro-enterologists and internal medicine physicians. A significant doctor’s delay may occur if these specialists are not familiar with the diagnosis of ACNES [6].

**Fig. 4:**
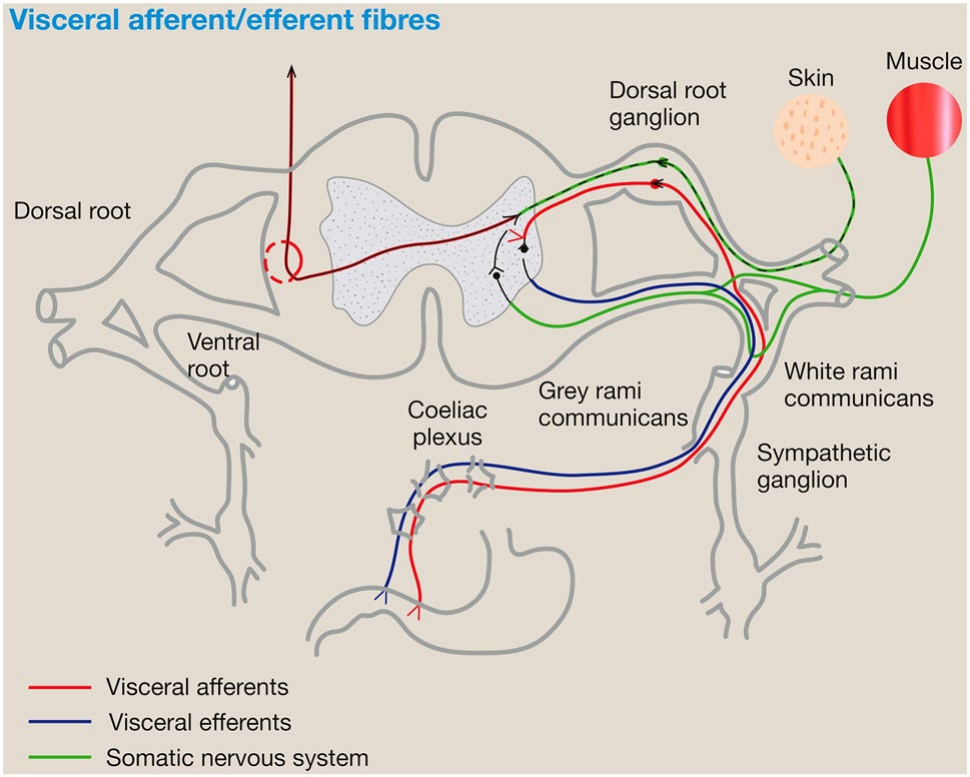
Illustration of the neuro-anatomy of visceral pain, taken from Kansal and Hughes [24]. Reprinted from Anaesthesia and Intensive Care Medicine, Vol. 20/ edition 10, Kansal A. and Hughes J., Visceral pain, Pages 550–554, Copyright (2019), with permission from Elsevier

The present study found that bloating was the most frequent visceral complaint in patients with ACNES, being reported in 78% of selected patients. Bloating, defined as the subjective sensation of abdominal distension, can affect from 10 to 30% of the general adult population and is often mentioned in patients with various gut disorders [18, 19]. Nevertheless, there is little available research on this topic. A review by Azpiroz and Malagelada provides an overview of the possible mechanisms of bloating. They hypothesized that bloating may be due to an abnormal *viscerosomatic* reflex involving abdominal distension and muscular wall dystonia [19]. A subsequent study showed this latter mechanism was responsible for a change in girth [8]. Viscerosomatic reflexes are also observed during other conditions, such as myocardial infarction causing pain radiating towards the arm, or in hepatobiliary disease that causes upper shoulder pain [20]. In 1907 already, Burns stated that “viscerosomatic reflexes would be impossible if viscero-sensory nerves did not enter the cord, or if they did not form either direct or indirect physiological relations with the somato-motor neuron” [21]. Moreover, the impulses should enter through the posterior roots of the spinal cord. Apart from viscerosomatic reflexes, *somatovisceral* reflexes also exist and have been hypothesized to explain the mechanism of action of sacral nerve stimulation in reducing fecal incontinence [22]. Bielefeldt et al. confirmed that multiple peripheral mechanisms (viscerosomatic, somatovisceral, and viscerovisceral convergence) lead to the development of hypersensitivity, thereby affecting neighboring organs or cutaneous referral sites [23]. Neuro-anatomy reveals that splanchnic nerves and somatic nerves are interconnected by the rami communicantes just before entering the spinal cord at the dorsal root (Fig. 4) [24]. This finding has led to the “convergence-projection theory”, which hypothesizes that dorsal horn neurons receive visceral input and convergent somatic input from the skin and/or muscle [25]. A recent in vitro thoracic spinal cord study showed that somatic and visceral C-fiber afferents converge mono-synaptically onto group lamina I neurons in the dorsal root [26]. These reflexes are also called segmental relations, reflecting the embryologic connection between various tissues including skin, muscles, bones and also the intestines that originate from the same segment. Therefore, based on the dorsal root reflex theory, the abdominal pain seen in ACNES may reflect such a disturbed segmental relationship [27]. Current daily medical practice with its emphasis on imaging and functional testing largely ignores the relevance of these phenomena.

We hypothesize that ACNES may result from a compromised segmental relation between a somatic (intercostal) nerve and the corresponding viscus, potentially resulting in a painful Head zone. In 1893, Sir Henry Head suggested that advanced visceral disease could lead to areas of altered skin sensation, with painful spots reflecting the (jeopardized) relation between a viscus and the corresponding somatic nerve dermatome [9]. Whereas the tender point in about half of ACNES patients is located in the right lower abdominal quadrant (T10–11), ACNES patients with a bariatric history are more prone to have a tender point in the left upper quadrant (T7–8) [28]. This latter type may be explained by a disturbed segmental relationship between the left 7–9th thoracic intercostal nerves and the splanchnic nerves innervating the stomach [29]. Furthermore, 40% of patients with acute appendicitis experienced altered skin sensations in the T10–11 right lower abdominal dermatome prior to appendectomy [10]. Interestingly, these changes were transient and disappeared postoperatively, suggesting that intercostal nerve pain may be associated with visceral disturbances and (temporary) loss or alteration of autonomic nerve system control.

A limitation of the present study is that the results are not applicable to all patients suffering from ACNES. Although a specific subgroup of ACNES patients was selected for this study, up to half of the general ACNES population may present with the same visceral symptoms [6]. Moreover, the size of the overall study cohort was relatively small, in particular the group with an unsuccessful response to treatment (*n* = 19). This study should, therefore, be regarded as descriptive and hypothesis generating. Another limitation involves the fact that VICAS is based on our own experience and requires further, independent validation. In addition, a possible relation between subjective treatment satisfaction and visceral symptoms was not studied. Since we only evaluated satisfaction in relation to pain reduction, patients may also have unintentionally considered a reduction (or not) in their visceral symptoms when reporting satisfaction for pain reduction. Future research should compare the characteristics of ACNES patients with and without visceral symptoms. To gain more insight into the origin of these symptoms in ACNES patients, it may be useful to compare the visceral symptoms of patients with ACNES to those of patients with other abdominal pathologies.

## Conclusion

A selected group of patients reporting abdominal pain concomitant with ACNES may experience a range of associated visceral symptoms. These symptoms can confuse the practitioner and lead to a prolonged search for a visceral origin of the pain, while overlooking a possible link to the abdominal wall. Adequate treatment for ACNES may reduce the pain, as well as the accompanying visceral symptoms. Therefore, ACNES should be included in the differential diagnosis of patients with visceral symptoms and abdominal pain. Further research is required on the clinical role of the involved, but often forgotten, segmental relationships between visceral and somatic innervation.

## Data Availability

The authors will consider data and video sharing requests on a case-by-case basis. Requests should be sent to the corresponding author for further information.
